# Development of a Caco-2-based intestinal mucosal model to study intestinal barrier properties and bacteria–mucus interactions

**DOI:** 10.1080/19490976.2024.2434685

**Published:** 2024-12-23

**Authors:** Evelien Floor, Jinyi Su, Maitrayee Chatterjee, Elise S. Kuipers, Noortje IJssennagger, Faranak Heidari, Laura Giordano, Richard W. Wubbolts, Silvia M. Mihăilă, Daphne A. C. Stapels, Yvonne Vercoulen, Karin Strijbis

**Affiliations:** a Center for Molecular Medicine, University Medical Center Utrecht, Utrecht University, Utrecht, The Netherlands; b Department of Biomolecular Health Sciences, Division of Infectious Diseases and Immunology, Faculty of Veterinary Medicine, Utrecht University, Utrecht, The Netherlands; c The TIM Company, Delft, the Netherlands; d Danone Research and Innovation Center, Utrecht, The Netherlands; e Div. Pharmacology, Utrecht Institute for Pharmaceutical Sciences, Utrecht University, Utrecht, The Netherlands

**Keywords:** Mucus, air–liquid growth, ALI, VIP, transwell, gut-on-a-chip, MUC2, tight junctions, intestinal mucosa, *Lactiplantibacillus plantarum*, ETEC, *Salmonella enterica* serovar Enteritidis

## Abstract

The intestinal mucosal barrier is a dynamic system that allows nutrient uptake, stimulates healthy microbe–host interactions, and prevents invasion by pathogens. The mucosa consists of epithelial cells connected by cellular junctions that regulate the passage of nutrients covered by a mucus layer that plays an important role in host–microbiome interactions. Mimicking the intestinal mucosa for *in vitro* assays, particularly the generation of a mucus layer, has proven to be challenging. The intestinal cell-line Caco-2 is widely used in academic and industrial laboratories due to its capacity to polarize, form an apical brush border, and reproducibly grow into confluent cell layers in different culture systems. However, under normal culture conditions, Caco-2 cultures lack a mucus layer. Here, we demonstrate for the first time that Caco-2 cultures can form a robust mucus layer when cultured under air–liquid interface (ALI) conditions on Transwell inserts with addition of vasointestinal peptide (VIP) in the basolateral compartment. We demonstrate that unique gene clusters are regulated in response to ALI and VIP single stimuli, but the ALI-VIP combination treatment resulted in a significant upregulation of multiple mucin genes and proteins, including secreted MUC2 and transmembrane mucins MUC13 and MUC17. Expression of tight junction proteins was significantly altered in the ALI-VIP condition, leading to increased permeability to small molecules. Commensal *Lactiplantibacillus plantarum* bacteria closely associated with the Caco-2 mucus layer and differentially colonized the surface of the ALI cultures. Pathogenic *Salmonella enterica* were capable of invading beyond the mucus layer and brush border. In conclusion, Caco-2 ALI-VIP cultures provide an accessible and straightforward way to culture an *in vitro* intestinal mucosal model with improved biomimetic features. This novel *in vitro* intestinal model can facilitate studies into mucus and epithelial barrier functions and in-depth molecular characterization of pathogenic and commensal microbe–mucus interactions.

## Introduction

The intestinal mucosa is a dynamic barrier tissue that allows the uptake of nutrients, provides an interaction surface for commensal microbiota, and prevents systemic pathogenic invasion. Intestinal epithelial cells are connected by adherence and tight junctions to form an organized monolayer with sufficient permeability. All intestinal epithelial cells express different types of highly glycosylated transmembrane mucins on their apical surface that together form the epithelial glycocalyx.^1^ Goblet cells within the epithelium produce and secrete gel-forming mucins that assemble into a viscous layer that covers the epithelium. The highly *O*-glycosylated MUC2 is the main soluble mucin of the intestinal tract. In the small intestine, the mucus layer is relatively thin to allow nutrient uptake, whereas the mucus layer of the large intestine consists of a sterile inner mucus layer and a more open outer mucus layer where the commensal microbiota reside and feed on the mucin glycans.^2^ Pathogenic bacteria have evolved virulence factors such as flagella and adhesins to breach the mucus layer and invade the epithelium. The mucus layer plays an important role in intestinal health, as exemplified by changes observed in inflammatory bowel diseases. During active disease, the number of goblet cells and MUC2 synthesis, secretion, or sulfation can be reduced, resulting in increased exposure of the epithelium to toxic agents and pathogens.^3–5^ In ulcerative colitis, the mucus layer is thinner compared to healthy individuals, while in Crohn’s disease, the quality of the mucus layer is altered, and fewer antimicrobials are present.^3^

*In vivo* or *ex vivo* models are available to study the function of the mucus layer, but these systems can be difficult to manipulate and do not always result in reproducible mucus production.^6^ *In vitro* intestinal cell cultures that include a mucus layer have also proven challenging to develop with both cell lines and primary culture models. There are reports of human enteroids and colonoids with good MUC2 production^7^ and human gut-on-a-chip primary cultures with a functional mucus layer.^8^ These cultures can also be used to study bacteria–mucus interactions, for example, with enteroaggregative *E. coli*^9^ or enterohemorrhagic *E. coli*^10^ or under anaerobic conditions using capped Transwells.^11^ The application of organoids for mucus research and studies into bacterial interactions with the intestinal epithelium was reviewed by Han et al.^12^ The challenge with primary human material is that culturing is costly, and cultures may not reach confluency simultaneously due to variable rates of cell division representative to the gastrointestinal tract. This often also results in differences between experiments. The Caco-2 cell line is a widely used model cell line in academic and industrial settings. Caco-2 cells grow robustly into confluent monolayers in regular culture medium, thereby reducing the costs of experiments. It is one of the few intestinal cell lines that is known to form a polarized epithelium and has been used in a coculture model with HT29-MTX cells to stimulate mucus production.^13^ For these reasons, we selected the Caco-2 cell line as a starting point to develop a mucus-coated intestinal epithelial model.

In primary intestinal material, it was previously demonstrated that a combination of air–liquid interface (ALI) culturing and the addition of vasointestinal peptide (VIP) could enhance mucus production.^14^ Transwell culture systems allow manipulation of both the apical and basolateral compartments, allowing ALI and liquid–liquid interface (LLI) culturing. In ALI culturing, cells are seeded and cultured to confluency on Transwell membranes, and the medium in the upper compartment is removed to expose the epithelial layer to air on the apical side and to liquid and nutrients on the basolateral side, more closely mimicking the *in vivo* situation compared to LLI culturing. It has been pointed out previously that ALI models are more suitable to model mucus properties.^15^

Gastrointestinal peptides such as VIP are critical regulators of intestinal functions and gut barrier. VIP was identified in porcine intestinal extracts in 1970^16^ and belongs to a superfamily of structurally related brain-gut peptide hormones.^17^ VIP can be produced by intrinsic enteric neurons in the mesenteric and submucous ganglionic plexuses, extrinsic parasympathetic autonomic nerves, and sensory fibers in the gut. In addition, some immune cells, such as lymphocytes, can also produce and secrete VIP (reviewed in^18^). Food consumption rapidly activates enteric neurons to express VIP.^19^ VIP was shown to activate receptors VPAC1, VPAC2, and PAC1, which are expressed by multiple cell types, including epithelial and immune, in the intestinal tract. VIP has a wide range of physiological effects on the intestinal tract, including regulation of smooth muscle relaxation for peristaltic movement in the upper GI tract, small and large intestine,^20–25^ secretion of luminal ions and fluid in the pancreas and jejunum,^26,27^ immune homeostasis^18^ and mucus production.^28^ In cultured colonic epithelial cells, which are known to express the VIP receptor VPAC1, VIP increased mucin production at both the mRNA and protein level.^28–31^ Moreover, a recent publication showed that the combination of ALI culturing and VIP could enhance mucus expression and secretion in primary intestinal material.^14^

In this study, we used the widely used intestinal Caco-2 cell line as a starting point and investigated the impact of ALI culturing and VIP on mucus production and epithelial barrier properties. We demonstrate that the combined ALI-VIP cultures lead to the production of a mucus layer consisting of both secreted and transmembrane mucins. In ALI-VIP conditions, expression of tight junctions was altered, leading to increased permeability to small molecules. Commensal and pathogenic bacteria demonstrated unique interactions with the mucus layer. In conclusion, our ALI-VIP Caco-2 intestinal mucosal model is easy-to-culture and robust. Moreover, it is suitable to study the function of the mucosa in microbe-host interactions, nutrient and drug uptake, and to assess intestinal epithelial barrier properties.

## Results

### Culturing under ALI-VIP conditions induces mucus production by Caco-2 cells

**Figure 1.**
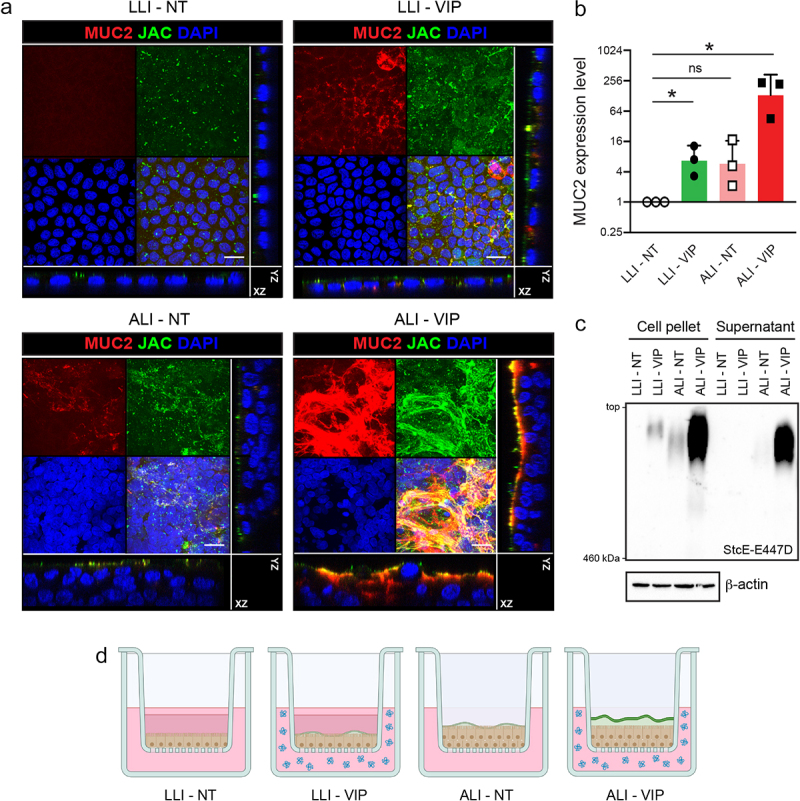
Combining air–liquid interface (ALI) and VIP treatment stimulates mucus production in caco-2 transwell cultures.

Caco-2 cells adapted to low-glucose culturing were grown in Transwell plates under four different low-glucose culture conditions, namely liquid–liquid interface non-treated (LLI-NT), liquid–liquid interface with VIP added to the basolateral compartment (LLI-VIP), air–liquid interface non-treated in which media was removed from the apical compartment (ALI-NT), and air–liquid interface with VIP added to the basolateral compartment (ALI-VIP). Under ALI culture conditions the Caco-2 cells formed a thicker multicellular layer compared to LLI culture conditions, where a monolayer of cells was formed (Figure 1a). To investigate if a mucus layer was produced under the different culture conditions the Transwell cultures were stained for the secreted intestinal mucin MUC2 and the glycoprotein-binding lectin Jacalin (JAC) and analyzed by immunofluorescence (IF) microscopy. In LLI-NT conditions, no MUC2 signal could be detected, while some MUC2 and JAC signal could be detected, which appeared in patches on the surface of LLI-VIP and ALI-NT cultures as evident from the orthogonal view. Interestingly, Caco-2 cells grown under ALI-VIP conditions showed prominent apical staining for both MUC2 and JAC, showing mucus layer formation on top of the cells (Figure 1a). qRT-PCR analysis confirmed that the MUC2 gene was highly upregulated in ALI-VIP conditions compared to LLI-NT (Figure 1b). To determine if the cellular layer formed by Caco-2 cells under the different culture conditions is polarized with an apical brush border, we stained the cell layers with villin, a marker for apical brush-border microvilli. Positive villin staining was detectable in the single-layer LLI cultures and the multilayer ALI cultures and did not seem to be significantly affected by VIP treatment (Fig. S1). To investigate if the detected MUC2 was secreted, we harvested the cellular material and supernatant fractions of all culture conditions. Lysates and supernatant fractions were analyzed using a mucin immunoblot protocol and stained with the StcE-E447D probe that can be used to detect *O*-glycosylated proteins. In line with the IF microscopy data, low levels of reactive material were detectable in the pellet fractions of LLI-VIP and ALI-NT conditions, and high levels of *O*-glycosylated protein was present in ALI-VIP cultures. Only in ALI-VIP cultures, the supernatant fraction contained a large amount of *O*-glycosylated material (Figure 1c). Spinning disk confocal microscopy was performed to further investigate the localization and secretion of MUC2 in the four different culture conditions. MUC2 and Jacalin staining were localized intracellularly in the Caco-2 cultures grown under LLI-NT, LLI-VIP, and ALI-NT conditions, while the ALI-VIP condition resulted in a MUC2 and Jacalin staining on the surface of the epithelium, very close to the villin signal (Fig. S1b, c). We zoomed in on MUC2, villin, and Jacalin staining in the orthogonal view of the ALI-VIP cultures. MUC2 and villin staining showed similar patterns, demonstrating a MUC2 localization very close to the apical brush border. The Jacalin staining pattern was distinct and localized most apically (Fig. S1d). These microscopy results did not allow a clear distinction between secreted or membrane-bound MUC2. However, we demonstrated above that secreted mucins are present in the supernatant fractions of the ALI-VIP cultures (Figure 1c). We hypothesize that some of the perceived differences in these assays relate to the washing steps necessary in the microscopy protocol that can remove unbound MUC2. We conclude that culturing our low-glucose adapted Caco-2 cells under ALI conditions with addition of VIP induces production and secretion of MUC2 and perhaps other mucins, resulting in the formation of an apical mucus layer (Figure 1d).

(a) Confocal microscopy of confluent low-glucose-adapted Caco-2 cultures grown on Transwell membranes in LLI and ALI conditions, not treated (NT) or treated with VIP. Cultures are stained for Jacalin (JAC, green), MUC2 (red), and nuclei (DAPI, blue). Maximum projections and orthogonal views are shown for the separate and combined channels. The white scale bar represents 20 μm. (b) qRT-PCR analysis of MUC2 expression in LLI-NT, LLI-VIP, ALI-NT, and ALI-VIP normalized to expression of ACTB. The graph displays the mRNA expression level relative to LLI-NT. Each dot represents a biological replicate. Statistical analysis was performed by a one-sample t-test. *p < .05 (c) Immunoblot analysis of mucin expression in the pellets and supernatant fractions of Caco-2 cells grown in LLI-NT, LLI-VIP, ALI-NT, and ALI-VIP conditions, visualized with the StcE-E447D probe which recognizes mucin-type O-glycosylated proteins. The top of the image corresponds to the loading slots of the agarose gel. The bottom of the image corresponds to the marker band of 460 kDa. (d) Schematic overview of Transwells containing Caco-2 cultures under LLI-NT, LLI-VIP, ALI-NT, and ALI-VIP conditions. LLI conditions contain medium in the top compartment, while the medium is removed in the ALI culture. Under LLI conditions, cultures are single-layered, while they become multilayered in ALI culture. VIP (indicated in blue) is added to the basolateral compartment. The highest mucus production is observed in the ALI-VIP combined conditions, which is the only condition showing clear apical mucus secretion (mucus layer indicated in green).

**Figure 2.**
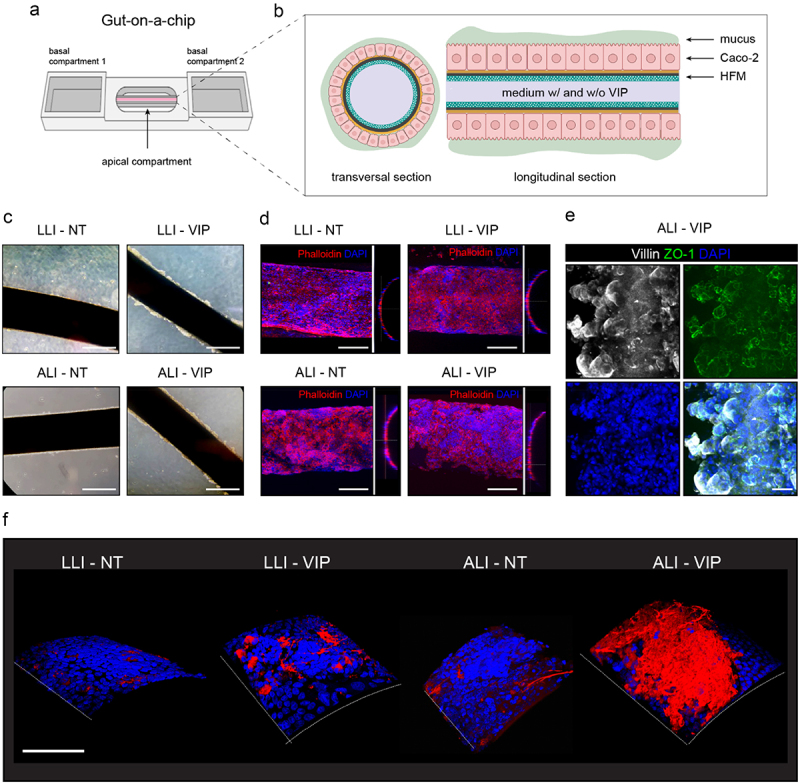
ALI-VIP conditions induce MUC2 production in a gut-on-a-chip model.

To assess reproducibility in alternative *in vitro* systems, the Caco-2 cells were grown in an adapted version of a previously described gut-on-a-chip model^32^ (Figure 2a,b). In short, cells were seeded on the outside surface of hollow fiber membranes (HFMs) that were placed in the inner chamber of the chips. 2-dimensional rocking of the chip allowed basolateral perfusion through the fiber interior by adding medium without or with VIP to the basolateral reservoir. In the apical reservoir of the chip, LLI and ALI conditions were applied as described above for the Transwells. Imaging of the cell-coated fibers demonstrated that the Caco-2 cells formed confluent layers on the surface of the fibers under all culture conditions (Figure 2c,d). VIP, ALI and combined ALI-VIP culturing induced a more multicellular organization, characterized by the formation of apical protrusions that are clearly visible in Figure 2c. These protrusions were reminiscent of villi-like structures that were previously observed in Caco-2 cultures on HFMs under flow conditions with shear stress.^32^ Confocal microscopy of HFMs coated with Caco-2 cells grown under ALI-VIP conditions demonstrated good staining for the brush border marker villin and the junction protein ZO-1 (Figure 2e and Supplementary movie 1). Staining for MUC2 showed enhanced production of MUC2 under ALI-VIP conditions and a moderate increase in MUC2 production under LLI-VIP conditions (Figure 2f). The gut-on-a-chip results corroborate the Transwell experiment and demonstrate a robust production of MUC2 by Caco-2 cells grown under ALI-VIP conditions in different systems.

(a) Gut-on-a-chip model consisting of a hollow fiber membrane (HFM) coated with Caco-2 cells mounted on a chip with 2 side reservoirs (the basolateral compartments) and one middle reservoir (the apical compartment). Cells are grown on the surface of the HFM and can be perfused basally through the fiber and apically via the inner compartment. (b) Schematic representation of the Caco-2 coated HFMs with formation of a secreted mucus layer. (c) HFMs coated with Caco-2 cells grown under LLI-NT, LLI-VIP, ALI-NT, and ALI-VIP conditions were captured using light microscopy. The white scale bar represents 500 μm. (d) Fluorescent confocal microscopy images of HFMs coated with Caco-2 cells grown under LLI-NT, LLI-VIP, ALI-NT, and ALI-VIP conditions stained for actin with phalloidin and nuclei with DAPI. The white scale bar represents 200 μm. (e) Fluorescent confocal microscopy of ALI-VIP HFM stained for villin, ZO-1, and DAPI. The white scale bar represents 50 μm. (f) 3-dimensional images of HFMs with Caco-2 cells stained for MUC2 (red) and nuclei (DAPI, blue). The white scale bar represents 200 μm.

### Transcriptional analysis of Caco-2 cultures

**Figure 3.**
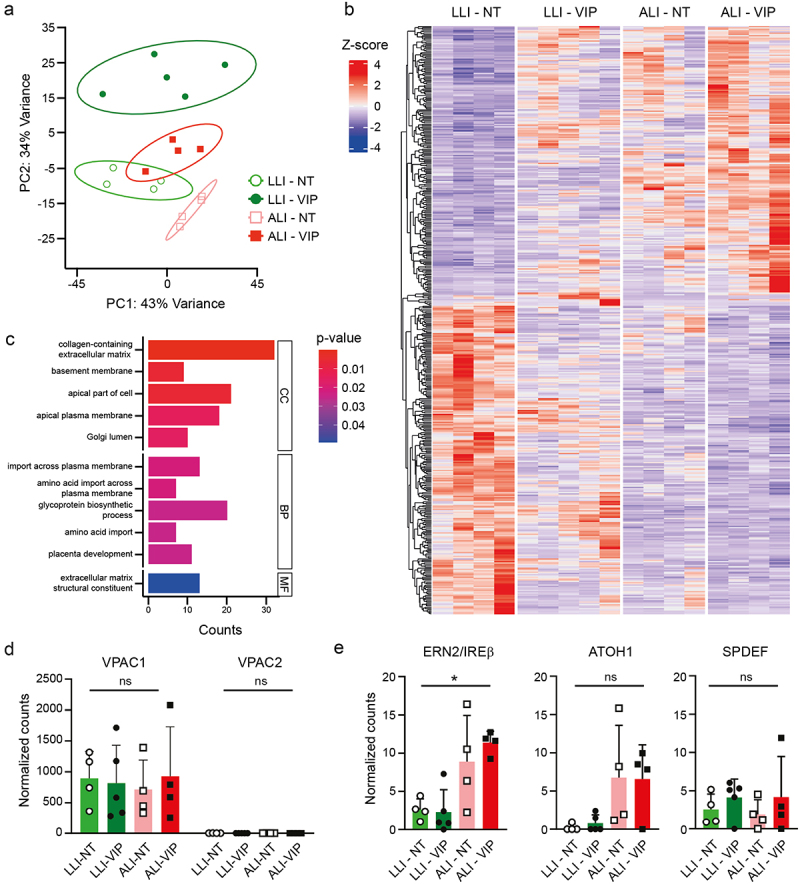
Transcriptomics analysis of differentiated Caco-2 cultures.

Next, we performed RNAseq analysis after 14 d of differentiation to investigate the impact of the different culture conditions on the total transcriptome. RNA was analyzed for 4–5 independent biological replicates of LLI-NT, LLI-VIP, ALI-NT, and ALI-VIP differentiated Caco-2 cells. A principal component analysis (PCA) of all samples showed distinct clusters of biological replicates per culture and condition (Figure 3a). A trend was visible that separated LLI from ALI culturing on one axis and VIP from no treatment on the other axis. Perhaps unexpectedly, the LLI-NT and ALI-VIP datasets clustered closest together. We next performed differentially expressed genes (DEG) analysis of the different treatments and used *p* < .05 as a cutoff for significantly up or downregulated genes. When taking the LLI-NT condition as the baseline, the ALI-VIP combined treatment yielded the largest set of 452 DEGs. ALI culturing, compared to LLI culturing, significantly altered the expression of 355 genes, while VIP addition changed the expression of 273 genes in the LLI condition and 49 in the ALI condition (Table 1). A full list of all DEGs of all culture conditions can be found in the supplementary materials (Table S1–5). The 452 significantly up- and downregulated genes between the ALI-VIP and LLI-NT conditions are presented as a heatmap (Figure 3b) and heatmaps with the gene labels (Fig. S2). In these heatmaps, noticeable variations between biological replicates of different culture conditions could be observed. We speculate that this diversity could be the result of tissue complexity after 14 d of differentiation and/or could, in part, be caused by different days of harvesting in our protocol. Gene Set Enrichment Analysis (GSEA) of the 452 DEGs in the ALI-VIP condition compared to the LLI-NT condition demonstrated that several cellular processes were significantly altered including import across plasma membrane, glycoprotein biosynthetic process and processes relating to the extracellular matrix and apical and basolateral membranes (Figure 3c). Overall, the gene expression profile of the Caco-2 cultures included several genes linked to small intestinal features such as CEACAM18 and MUC17. Expression of WNT5A and lysozyme expression was comparable between all conditions. ALI-VIP culturing did induce some changes in expression of marker genes, but the overall profile remained similar between growth conditions (Fig. S3a). Therefore, we conclude that the basic features of Caco-2 cells were retained during ALI-VIP culturing.

(a) PCA plot of full RNAseq datasets derived from 4 to 5 replicates of the Caco-2 LLI-NT, LLI-VIP, ALI-NT, and ALI-VIP conditions. (b) Heatmap of the 452 differentially expressed genes (DEGs) between the LLI-NT and ALI-VIP conditions. See also Figure S2 for heatmaps of up- and down-regulated genes with gene labels and supplementary tables S1-S5 for a full list of all DEGs between the different culture conditions. (c) Gene Set Enrichment Analysis (GSEA) of the 453 DEGs in the ALI-VIP condition compared to the LLI-NT condition in the GO clusters cellular compartment (CC), biological processes (BP), and molecular function (MF). (d) Expression of VPAC1 and VPAC2 in the RNAseq dataset. Normalized counts for individual data points of the biological replicates are plotted. (e) Expression of mucus-related transcription factors ERN2/IRE1β, ATOH1, and SPDEF in the RNAseq dataset. Normalized counts for individual data points of the biological replicates are plotted. Statistical analysis was performed by ordinary one-way ANOVA under mixed-effect analysis using GraphPad Prism software (v10.1.1). *p < .05.

**Table 1.** DEGs in different conditions.

Comparison	Number of DEGs	Effect
LLI-VIP to LLI-NT	273	VIP effect in LLI
ALI-NT to LLI-NT	355	ALI effect
ALI-VIP to ALI-NT	49	VIP effect in ALI
ALI-VIP to LLI-VIP	200	ALI effect in VIP
ALI-VIP to LLI-NT	452	ALI-VIP combined effect

The VIP receptor VPAC1 was found to be highly expressed by the Caco-2 cells, and expression was stable across the different culture conditions, whereas expression of VPAC2 was relatively low (Figure 3d). To understand how ALI-VIP culturing could induce mucus production in Caco-2 cells, we analyzed the expression levels of transcription factors and regulators previously implicated in differentiation of goblet cells and/or expression of mucins. From the RNAseq data, we plotted the expression of ERN1/IRE1α ERN2/IRE1β, XBP1, AGR2, ATF4, ATF6, PERK, ATOH1, TFF3, SPDEF and CDX2 in the LLI-NT, LLI-VIP, ALI-NT and ALI-VIP samples (Fig. S3b). Of these transcription factors, only the intestinal epithelial-specific ER stress sensor ERN2/IRE1β was significantly upregulated in the ALI-VIP condition compared to LLI-NT (Figure 3e). The ERN2/IRE1β sensor is essential for goblet cell differentiation and allows adaptation of the unfolded stress response in the ER to allow for production of MUC2 in goblet cells.^33,34^ The transcription factors ATOH1 and SPDEF are regulators of intestinal differentiation and goblet cell formation and are responsible for the regulation of a network of genes involved in mucus production.^35–37^ In our dataset, ATOH1 showed a non-significant trend that indicated upregulation under ALI conditions. SPDEF was perhaps slightly upregulated in the VIP conditions, but the expression was highly variable between replicates (Figure 3e). Based on these data, we speculate that a combination of upregulation of important transcription factors in the ALI-VIP condition results in successful expression and secretion of mucus.

### Multiple mucin proteins are upregulated in Caco-2 ALI-VIP cultures

**Figure 4.**
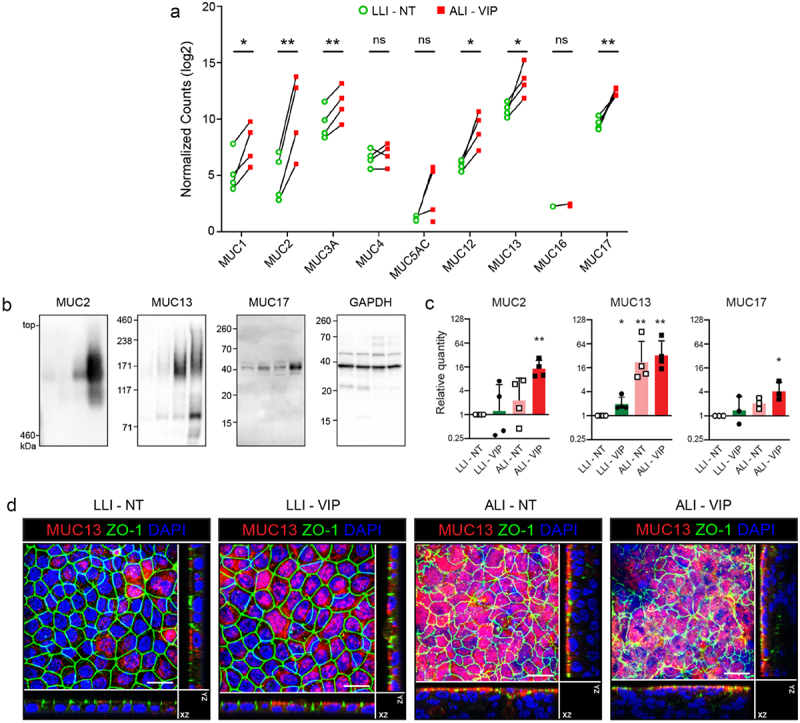
Multiple mucins are upregulated under ALI-VIP conditions.

Within the 30 most highly upregulated genes in the ALI-VIP condition, we noticed that a high number of mucin genes ranked amongst the most highly upregulated genes (Table 2), of which MUC2 was the second most differentially expressed gene. Moreover, secreted mucin MUC5AC and transmembrane mucins MUC12, MUC13, and MUC17 were amongst the most highly upregulated genes. From the complete dataset, we selected known mucin genes MUC1, MUC2, MUC3A, MUC4, MUC5AC, MUC12, MUC13, MUC16, MUC17, and MUC20 and plotted their expression in matched samples grown under LLI-NT and ALI-VIP conditions. MUC1, MUC2, MUC3A, MUC5AC, MUC12, MUC13, and MUC17 were significantly upregulated in the ALI-VIP conditions (Figure 4a). A comparison of the four culture conditions demonstrated that the addition of VIP-induced mucin expression (Fig. S3C). Next, we determined protein expression of selected mucins in the different culture conditions by immunoblot (Figure 4b). Quantification of the immunoblot data demonstrated that MUC2 and MUC17 were significantly upregulated in ALI-VIP conditions and that MUC13 was significantly upregulated in both ALI-NT and ALI-VIP conditions (Figure 4c). We previously demonstrated that the transmembrane mucin MUC13 is a negative regulator of intestinal epithelial tight junctions.^38^ Therefore, we performed immunofluorescence microscopy on the Caco-2 cultures and observed an increase in MUC13 expression on the apical membrane and reduced intensity of ZO-1 staining patterns under ALI-VIP conditions (Figure 4d). Together, these results demonstrate that several mucin genes are upregulated under ALI-VIP culture conditions and that while mucus production is elevated, the tissue morphology and cellular junctions are altered, suggesting an effect on epithelial barrier function.

(a) Expression of mucin genes in the RNAseq dataset. Normalized counts for individual data points of the biological replicates are plotted as paired sets for matched biological samples from the same experiment. Statistical analysis was performed using transformed data by a two-tailed paired t-test using GraphPad Prism software (v10.1.1). *p < .05; **p < .01. (b, c) Immunoblot analysis and quantification of MUC2, MUC13, and MUC17 relative to expression of GAPDH in LLI-NT, LLI-VIP, ALI-NT and ALI-VIP conditions. Each dot represents a biological repeat. Statistical analysis was performed by a one-sample t-test. (d) Confocal microscopy analysis of MUC13 and ZO1 expression in the indicated Caco-2 culture conditions. Maximum projections and orthogonal views are shown. The white scale bar represents 50 μm.

**Table 2.** Top 30 upregulated genes in ALI-VIP conditions compared to LLI-NT.

Gene symbol	FC (log2)	P adjusted	Gene name
REN	8,51	5,0E-06	renin
MUC2	6,58	1,8E-03	mucin 2, oligomeric mucus/gel-forming
AKAP4	5,94	2,1E-02	A-kinase anchoring protein 4
SLC18A1	5,83	4,2E-03	solute carrier family 18 member A1
lncRNA (NA)	5,56	2,3E-02	Lnc-DYNLRB2-3
LGALS7	5,55	4,7E-03	galectin 7
lncRNA (NA)	5,48	1,8E-02	Lnc-FGGY-6
CEACAM5	3,94	9,7E-03	CEA cell adhesion molecule 5
MUC5AC	3,86	4,0E-02	mucin 5AC, oligomeric mucus/gel-forming
GALR2	3,75	4,1E-02	galanin receptor 2
SLC6A20	3,68	6,4E-09	solute carrier family 6 member 20
SSTR5-AS1	3,65	1,6E-03	SSTR5 antisense RNA 1
MUC12	3,64	5,1E-04	mucin 12, cell surface associated
CTRC	3,52	3,7E-04	chymotrypsin C
ST6GALNAC1	3,42	3,2E-03	ST6 N-acetylgalactosaminide alpha-2,6-sialyltransferase 1
MANCR	3,41	3,9E-02	mitotically associated long non-coding RNA
FIBCD1	3,40	2,4E-02	fibrinogen C domain containing 1
TRIM50	3,39	1,6E-04	tripartite motif containing 50
KRTAP5-AS1	3,23	2,7E-02	KRTAP5-1/KRTAP5-2 antisense RNA 1
GJB4	3,21	6,4E-05	gap junction protein beta 4
GGT6	3,07	1,1E-03	gamma-glutamyltransferase 6
MUC13	3,02	7,4E-04	mucin 13, cell surface associated
CLCNKA	3,02	1,0E-02	chloride voltage-gated channel Ka
B3GNT3	2,99	3,6E-05	UDP-GlcNAc:betaGal beta-1,3-N-acetylglucosaminyltransferase 3
SPNS2	2,94	2,4E-02	sphingolipid transporter 2
C9orf152	2,88	3,2E-05	chromosome 9 open reading frame 152
CAPN11	2,80	4,1E-02	calpain 11
MUC17	2,76	5,3E-10	mucin 17, cell surface associated
TNFSF15	2,72	3,9E-02	TNF superfamily member 15
PLA2G2A	2,67	3,3E-06	phospholipase A2 group IIA

### Tight junction composition is altered in caco-2 ALI-VIP cultures, leading to increased permeability to small tracers

**Figure 5.**
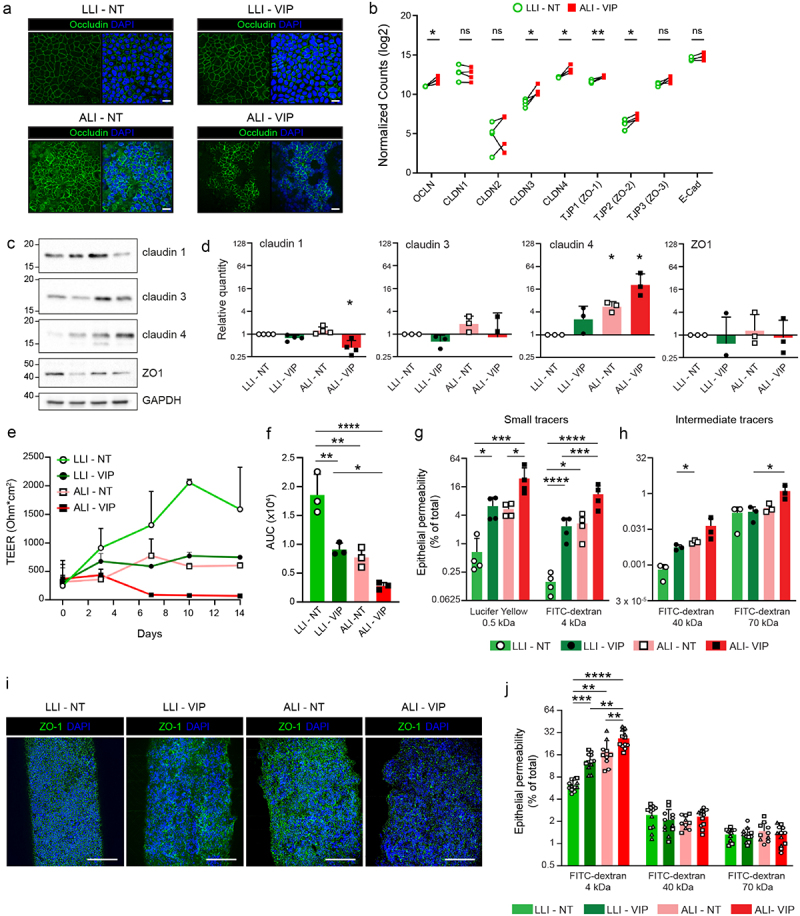
Tight junction composition is altered, and epithelial permeability increased under ALI-VIP conditions.

To continue investigating the cellular junctions in our different Caco-2 cultures, we stained for the main tight junction (TJ) protein occludin. In line with the ZO-1 staining in Figure 4d, we again observed very organized TJ staining in the single-layered LLI culture, while the TJ staining pattern in the multilayered ALI cultures was more irregular (Figure 5a). Similar to the analysis of the mucin genes, we extracted expression data for tight and adherence junction genes from our RNAseq dataset. Matched LLI-NT and ALI-VIP samples demonstrated that *OCLN*, *CLDN3*, *CLDN4*, *TJP1* (*ZO-1*) and *TJP2* (*ZO-2*) were significantly upregulated (Figure 5b). The expression of other genes encoding junction proteins was relatively comparable between the four conditions (Fig. S3d).

(a) Confocal microscopy of different Caco-2 cultures stained for occludin demonstrating altered organization of tight junctions in the ALI cultures compared to the LLI cultures. The white scale bar represents 20 μm. (b) Expression of genes related to tight junctions in the RNAseq dataset. Normalized counts for individual data points of the biological replicates are plotted as paired sets for matched biological samples from the same experiment. Statistical analysis was performed using transformed data by a two-tailed paired t-test. *p < .05; **p < .01. (c,d) Immunoblot analysis and quantification of claudin-1, claudin-2, claudin-4, and ZO-1 relative to expression of GAPDH in LLI-NT, LLI-VIP, ALI-NT and ALI-VIP conditions. Statistical analysis was performed by a one-sample t-test. (e) Transepithelial electrical resistance (TEER) assay for Caco-2 LLI-NT, LLI-VIP, ALI-NT, and ALI-VIP cultures from the start of differentiation, e.g., day 3 of cell culture, referred to as day 0 over a course of 14 d. (f) Area under the curve (AUC) measurements of experiments shown in E. Statistical analysis was performed by ordinary one-way ANOVA using GraphPad Prism software. *p < .05; **p < .01; ****p < .0001. (g) Tissue permeability measurements with Transwell Caco-2 cultures with small tracers lucifer yellow (0.5 kDa) and FITC-Dextran 4 kDa particles under the indicated culture conditions. Tracer amounts in the basolateral compartment were quantified and expressed as percentage of maximum permeability. Data were transformed, and statistical analysis was performed by ordinary one-way ANOVA. *p < .05; **p < .01. (h) Experiment as in G with intermediate-sized tracers FITC-Dextran 40 kDa and 70 kDa. (i) Confocal microscopy of different gut-on-a-chip HFM Caco-2 cultures stained for ZO-1. The white scale bar represents 200 μm. (j) Tissue permeability measurements with gut-on-a-chip HFM Caco-2 cultures with FITC-Dextran 4 kDa, 40 kDa, and 70 kDa particles under the indicated culture conditions. Combined tracer amounts from both basolateral compartments were quantified and expressed as percentage of maximum permeability. The three different symbols correspond to chips measured as part of three independent experiments. Data were transformed, and statistical analysis was performed by ordinary one-way ANOVA under mixed-effect analysis. *p < .05; **p < .01; ***p < .0001; ****p < .0001.

Claudins can be barrier-forming or pore-forming and together regulate the paracellular transfer of small molecules across the intestinal epithelial barrier. We have previously shown that MUC13 negatively regulates claudin-1, claudin-3, and claudin-4 expression, leading to increased tight junction strength in the absence of MUC13.^38^ We therefore analyzed claudin expression in more detail by immunoblot (Figure 5c). Quantification demonstrated that claudin-1 was significantly downregulated in ALI-VIP conditions, while claudin-4 was significantly upregulated in both ALI conditions. No significant change in expression was observed for claudin-3 and ZO-1 (Figure 5d). To determine how these changes in expression of TJ proteins influence intestinal epithelial barrier properties, we first measured the transepithelial electrical resistance (TEER). Caco-2 cells grown in LLI-NT conditions built a high TEER to 2000 Ohm*cm^2^. In all the other conditions, a lower TEER was observed that was most significant in the ALI-VIP cultures (Figure 5e,f). Next, we investigated the transfer of small and intermediate-sized tracer molecules across the epithelial layer under different growth conditions. The transfer of small tracers lucifer yellow (0.5 kDa) and FITC-Dextran 4 kDa particles to the basolateral compartment was significantly enhanced by the addition of VIP and ALI culturing. The combined ALI-VIP condition demonstrated the highest permeability to both small tracer molecules (Figure 4g). For tracers of intermediate size (FITC-Dextran particles of 40 and 70 kDa), the difference between conditions was not statistically different between LLI-NT and ALI-VIP conditions (Figure 5h). We also used the gut-on-a-chip system to investigate the permeability of the Caco-2 cultures under different growth conditions. First, we stained the Caco-2 cultures on the fibers for the junction protein ZO-1. Cellular junctions were visible in all culture conditions (Figure 5i). Experiments with the small 4 kDa FITC-Dextran tracer demonstrated a stepwise enhanced permeability of LLI-VIP, ALI-NT and ALI-VIP gut-on-a-chip cultures (Figure 5j), corroborating the results with the Transwells. The permeability to intermediate tracers of 40 and 70 kDa was comparable between culture conditions. In conclusion, under ALI-VIP conditions, Caco-2 cells produce a secreted mucus layer, upregulate different mucin proteins and expression of different junction proteins is altered, resulting in increased permeability of the epithelial barrier to small molecules.

### Application of the Caco-2 mucus model in bacterial colonization and infection experiments

**Figure 6.**
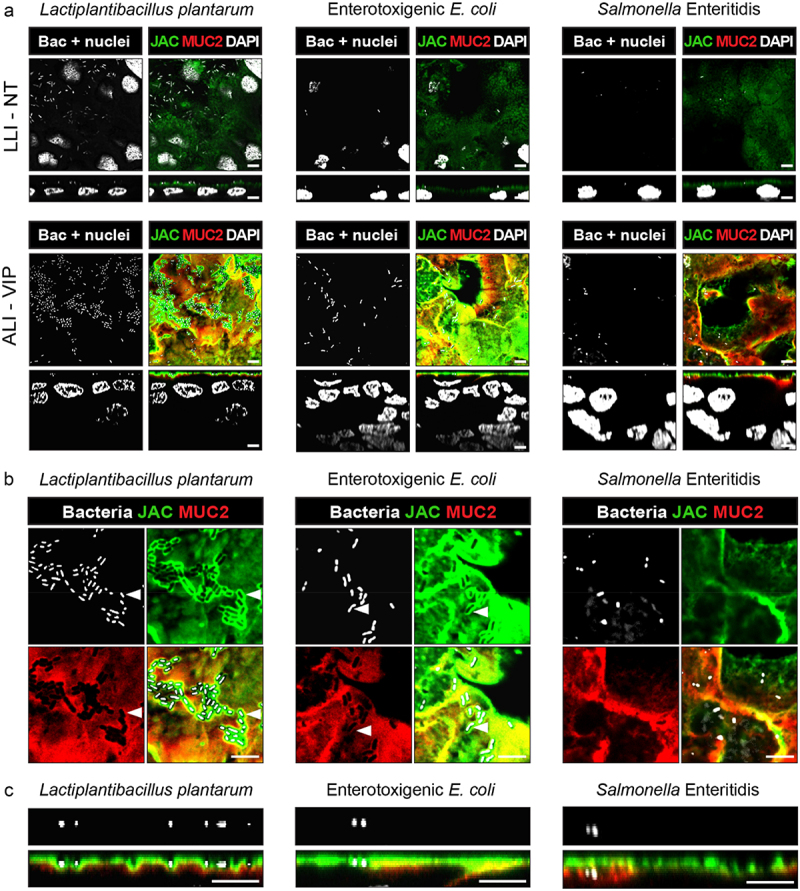
Infection experiments with different bacteria demonstrate unique interactions with the ALI-VIP mucus layer.

We next investigated if our novel Caco-2 mucus model could be used to study interactions of commensal and pathogenic bacteria with the intestinal mucus layer and underlying epithelium. Caco-2 cells were grown in LLI-NT and ALI-VIP conditions followed by incubation with the commensal bacterium *Lactiplantibacillus plantarum*, pathogenic enterotoxigenic *E. coli* (ETEC) or *Salmonella enterica* Enteritidis under aerobic conditions. Both pathogens were incubated with the tissues for 1 h, as we have previously demonstrated that invasion can occur in this time frame.^39^ For the highly invasive *Salmonella* Enteritidis, 10× less bacteria were used. *L. plantarum* was incubated with the tissues for 3 h to allow commensal colonization. After infection, tissues were stained for MUC2 and Jacalin to visualize the mucus layer and DAPI to visualize both bacteria and epithelial nuclei. As in previous experiments, a thicker multilayered epithelium formed under ALI-VIP conditions, and a MUC2-positive layer close to the epithelium and Jacalin staining on top could be observed (Figure 6a). All three bacteria were adhering to the ALI-VIP cultures, and *L. plantarum* also showed extensive adhesion to the LLI-NT cultures (Figure 6a). In a larger magnification, we observed that *L. plantarum* and ETEC localized to the Jacalin layer and that Jacalin staining was enriched around the bacteria (Figure 6b, left and middle). Some enrichment of MUC2 signal could also be observed around *L. plantarum* and ETEC. From the orthogonal views, we observed that there was an enrichment in Jacalin staining around the *L. plantarum* bacteria, suggesting their association with the apical interaction surface of the secreted mucus layer (Figure 6c, left). A similar enrichment in Jacalin signal was observed around the ETEC bacteria (Figure 6c, middle). For the *Salmonella* Enteritidis bacteria, on the other hand, no enrichment of Jacalin or MUC2 signal was observed around the bacteria (Figure 6b, right). In the orthogonal views, *Salmonella* Enteritidis bacteria could be observed below the Jacalin layer, which is in line with the invasive properties of this bacterium (Figure 6c, right).

(a) Confocal microscopy of infection experiments with LLI-NT (top) or ALI-VIP (bottom) Caco-2 Transwell cultures with *Lactiplantibacillus plantarum* for 3 h, enterotoxigenic *E. coli* (ETEC) for 1 h, or *Salmonella* Enterica for 1 h. XY plane on top displaying the Z-level with the highest concentration of bacteria, XZ plane below. Bacteria and Caco-2 nuclei are stained with DAPI (white), and the mucus layer is stained with Jacalin (green) and MUC2 (red). Scale bars represent 5 μm. (b) Enlarged view of ALI-VIP cultured Caco-2 cells infected with *Lactiplantibacillus plantarum*, enterotoxigenic *E. coli* (ETEC), or *Salmonella* Enterica or from panels in (a) visualized in the XY plane. (c) Enlarged view of ALI-VIP cultured Caco-2 cells infected with *Lactiplantibacillus plantarum*, enterotoxigenic *E. coli* (ETEC), or *Salmonella* Enterica visualized in the XZ plane from panels in (a). White scale bars represent 5 μm.

**Figure 7.**
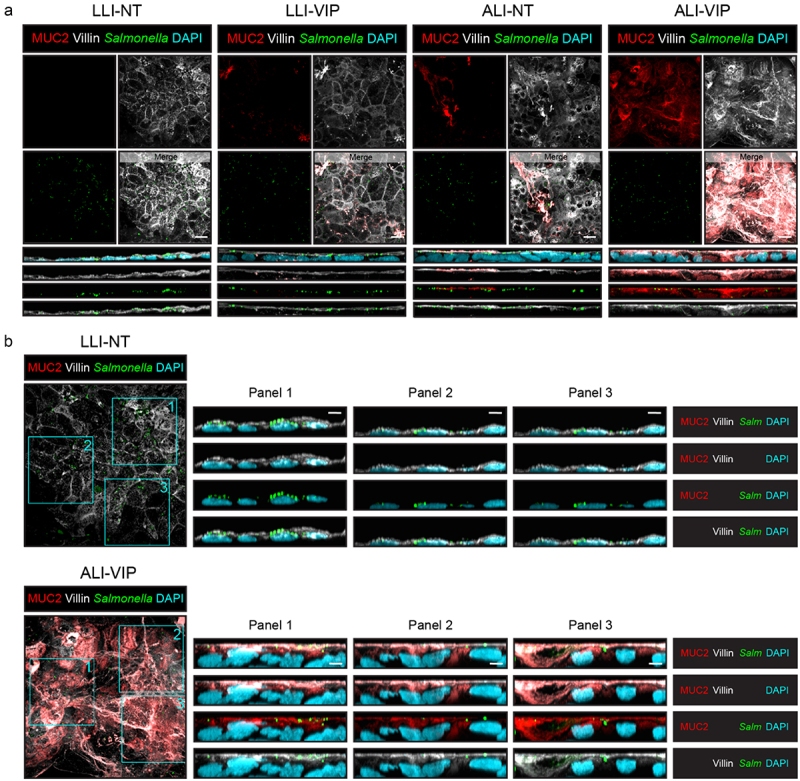
*Salmonella* invades the Caco-2 ALI-VIP epithelium beyond the mucus layer.

To investigate *Salmonella* Enteritidis invasion into the Caco-2 mucus model in more detail, we performed infection experiments with a fluorescent *Salmonella* Enteritidis with Caco-2 cultures grown under all four culture conditions. Cultures were stained for MUC2 and villin to visualize the position of the bacteria relative to the mucus layer and apical brush border. Adherence of GFP-positive bacteria to the epithelium was observed for all four culture conditions after 1 h of infection (Figure 7a). In both the LLI-NT and ALI-VIP conditions, the bacteria localized in the vicinity of the villin-positive brush border. In the orthogonal view of the ALI-VIP cultures, bacteria could be observed below the brush border, in part facilitated by the enhanced thickness of the ALI-VIP cultures (Figure 7b).

(a) Confocal microscopy of infection experiments with LLI-NT, LLI-VIP, ALI-NT, and ALI-VIP Caco-2 Transwell cultures infected with a GFP-positive *Salmonella enterica* serovar Enteritidis strain for 1 h. Cultures were stained for MUC2 (red; the mucus layer), villin (white; the apical membrane), and DAPI (blue; nuclei). Orthogonal views display the relative proximity of the bacteria to the mucus layer and apical membrane. The order of the displayed channels is the same as listed for B. White scale bars represent 10 μm. (b) Enlarged view of *Salmonella*-GFP infection of LLI-NT and ALI-VIP cultures, including XZ plane orthogonal views of three areas (Panel 1–3). Staining of samples as in (a). White scale bars represent 5 μm.

**Figure 8.**
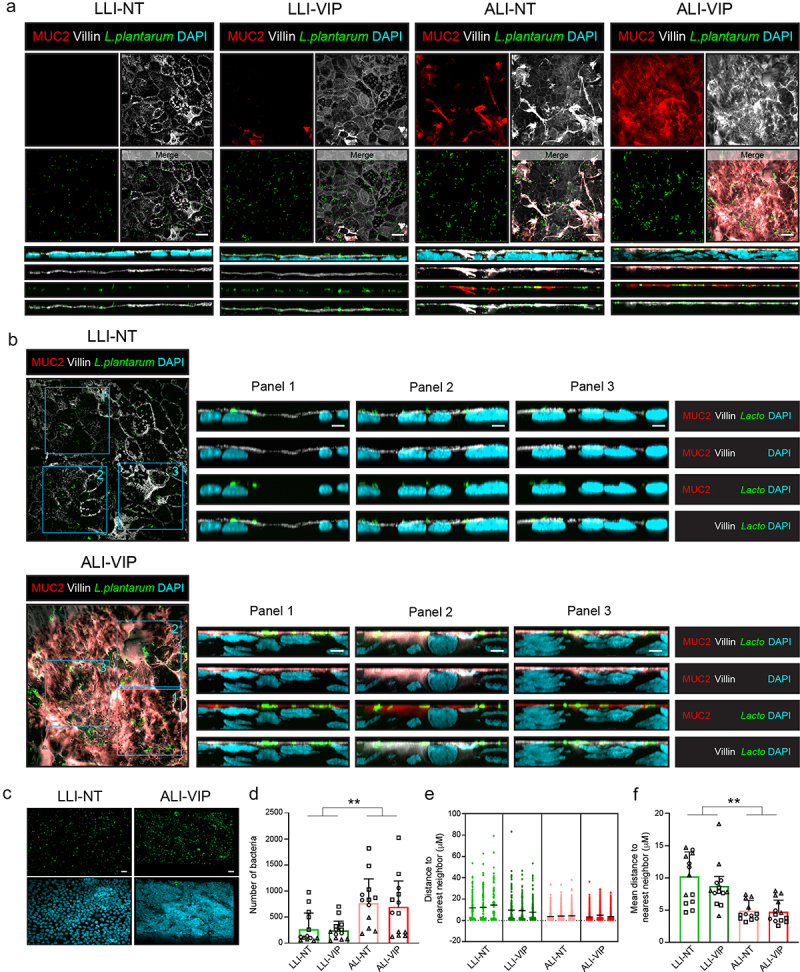
Colonization of Caco-2 cultures by *Lactiplantibacillus plantarum.*

Next, we looked in more detail at the interaction of *Lactiplantibacillus plantarum* with the ALI-VIP mucus layer. Caco-2 cultures were incubated with *Lactiplantibacillus plantarum* for 1 h, after which tissues were stained for MUC2 and villin. Bacteria adhered to the surface of all four Caco-2 cultures (Figure 8a). Orthogonal views of the LLI-NT and ALI-VIP condition demonstrated that the bacteria remained associated with the apical surface and the mucus layer in the ALI-VIP condition (Figure 8b). In the LLI-NT condition, single bacteria were visible on the surface, while bacterial clusters could be observed in the ALI-VIP condition (Figure 8b, left). Imaris software was used to mask the bacteria (Figure 8c), quantify them in the four culture conditions, and characterize their clustering behavior on the surface. Under ALI conditions, more bacteria adhered to the Caco-2 cultures (Figure 8d). Since bacterial clustering demonstrates adaptation to the mucosal nice, we quantified clustering by determining for each individual bacterium their nearest neighbor (Figure 8e). In both ALI culturing conditions, independent of VIP addition, significantly higher clustering of the *Lactobacilli* was observed. We conclude that *L. plantarum* and ETEC closely interact with the ALI-VIP mucus layer and that invasive *Salmonella* Enteritidis is capable of passing this mucus layer. These infection and colonization experiments demonstrate that the Caco-2 ALI-VIP mucus model can be used as a platform with biomimetic features to investigate interactions of commensal and pathogenic bacteria with the intestinal mucus layer.

(a) Confocal microscopy of colonization experiments of LLI-NT, LLI-VIP, ALI-NT, and ALI-VIP Caco-2 Transwell cultures incubated with CFSE-labeled *Lactiplantibacillus plantarum* (green) for 1 h. Cultures were stained for MUC2 (red; the mucus layer), villin (white; the apical membrane), and DAPI (blue; nuclei). Orthogonal views display the relative proximity of the bacteria to the mucus layer and apical membrane. The order of the displayed channels is the same as listed for B. White scale bars represent 10 μm. (b) Enlarged view of *L. plantarum*-CFSE colonization of LLI-NT and ALI-VIP cultures, including the maximum project of 10 slides XZ plane orthogonal views of three areas (Panel 1–3). Staining of samples as in (a). White scale bars represent 5 μm. (c) Representative LLI-NT and ALI-VIP images of fluorescent bacteria used for masking and quantification. White scale bars represent 20 μm. (d) Quantification of the number of attached bacteria per condition. Different symbols represent three independent biological replicates, of which 3–5 fields were quantified. Data were grouped by LLI or ALI culture, and statistical analysis was performed by a two-tailed paired t-test. *p < .05; **p < .01. (e) Plot displaying quantification of distance of a bacterium to the nearest neighboring bacterium. Quantification of a single field of each of the four culture conditions is shown as representative analysis. (f) The quantification of clustering of bacteria based on distance to the nearest bacteria. Different symbols represent three independent biological replicates, of which 3–5 fields were quantified. Data were grouped by LLI or ALI culture, and then statistical analysis was performed by a two-tailed paired t-test *p < .05; **p < .01.

## Discussion

The mucus layer is an essential component of the intestinal mucosal epithelium, but many *in vitro* intestinal epithelial cultures lack a mucus layer. The use of primary models such as intestinal organoids is increasing. However, these cultures are expensive and do not always include a mucus layer. Culturing of the widely used Caco-2 intestinal cell line is more straightforward and reproducible, but these cultures lack a mucus layer. In this study, we present for the first time that Caco-2 can be cultured in such a way that an apical mucus layer is consistently produced within 14 d after cell confluency on the Transwell. We took our inspiration from a previous study with primary intestinal material^14^ and investigated the effects of ALI culturing and addition of VIP on mucus production and epithelial barrier properties in Caco-2 cultures grown on Transwell membranes for 14 d. This novel *in vitro* Caco-2-based mucosal model can be used to study intestinal barrier functions and microbe–mucus interactions.

The Caco-2 cells were grown on Transwell plates in low-glucose medium. Our experiments demonstrated that the single stimuli (addition of VIP under LLI conditions or ALI culturing without VIP) induced unique changes in gene and protein expression and epithelial morphology. However, the ALI-VIP combined treatment resulted in the strongest effect in which a robust and functional mucus layer was produced. In addition to the robust mucus layer in the Caco-2 ALI-VIP tissue, we observed altered organization and composition of tight junctions and increased permeability of the tissues for small tracers but not for larger molecules. Commensal *L. plantarum* bacteria closely interacted with the ALI-VIP mucus layer and formed dense clusters in both types of ALI cultures that remained on the apical surface. Pathogenic *Salmonella*, on the other hand, were capable of invading the epithelium beyond the mucus layer.

Our data demonstrate that the VIP peptide and ALI culturing synergize and are both required to fully induce MUC2 secretion mucus layer formation (Figure 1a, Figure S1b,c). While ALI culturing did lead to a high number of DEGs (Table 1) and induction of the ERN2/IRE1β system, addition of VIP in the ALI condition only significantly alters an additional 49 genes, and some of these could be essential for optimal mucus formation. However, this could also point toward a potential posttranscriptional effect of VIP that facilitates full expression, assembly, and secretion of MUC2 by Caco-2 cells. An interesting future direction would be to investigate the porosity and viscosity of the Caco-2 mucus layer, how it relates to mucus properties in vivo, and if VIP also plays a role in regulating mucus characteristics.

The vasoactive intestinal peptide has a wide range of physiological functions in the intestine and provides a link between goblet cell differentiation, mucus production, and microbiota composition. For example, VIP increased the number of ileal goblet cells in mice^40^ and regulated the commensal microbiota composition.^19^ In cultured colonic epithelial cells, which are known to express the VIP receptor VPAC1, VIP increased mucin production at both the mRNA and protein level.^28–31^ The transcription factor Cdx2 regulates proliferation, differentiation, and migration of intestinal epithelial cells was previously implicated in regulating VIP-mediated MUC2 increase.^41^ In our dataset, high expression of VPAC1 is observed, but CDX2 mRNA expression is not induced by VIP (Figure 2). We do observe a trend of upregulation of the SPDEF transcription factor, a master regulator of mucin-related genes in response to VIP. Culturing in air–liquid interface conditions has been applied widely to respiratory epithelial cultures (reviewed in ^42,43^). It has been previously demonstrated that ALI culturing triggers differentiation and a metabolic shift in porcine intestinal cells.^44–46^ Our RNAseq analysis demonstrates that ALI conditions stimulate expression of the intestinal epithelial-specific ER stress sensor ERN2/IRE1β (Figure 2). ERN2/IRE1β induces XBP1 splicing and regulates transcription of genes involved in the unfolded protein response that facilitates synthesis and secretion of large quantities of proteins such as MUC2.^33^ In line with our observations, studies with ERN2 and IRE1β knockout mice showed that goblet cell induction or MUC2 expression, respectively, is abolished in the absence of these sensors.^33,34^

Under ALI-VIP conditions, the Caco-2 Transwell culture is multilayered and produces a robust mucus layer.

The morphology and composition of tight junctions are also altered in ALI-VIP conditions, and significant changes in expression of claudin-1 and claudin-4 are observed compared to the LLI-NT condition. In conjunction with these changes, we observe that the ALI-VIP Caco-2 layer is more permeable, as can be seen by the low TEER and increased transfer of small tracers across the tissue (Figure 5). TEER values of gastrointestinal epithelia are considered “tight” at values of about 2000 Ohm*cm^2^, intermediate around 300–400 Ohm*cm^2^ and leaky around 50–100 Ohm*cm^2^ .^47^ The latter values are corresponding to TEER properties of human small intestinal epithelium.^48^ It was previously demonstrated that under normal culture conditions, TEER values of Caco-2 monolayers can range between 200 and 1000 Ohm*cm^2^ which reflects a permissive epithelium. In our experiments, TEER values of the LLI-NT, LLI-VIP and ALI-NT conditions fall within the 200–1000 Ohm*cm^2^ range, while under ALI-VIP conditions, TEER was reduced to 50–100 Ohm*cm^2^. This lower TEER might again point in the direction of enhanced small-intestinal features of the ALI-VIP cultures. In addition, the presence of Goblet cells was previously suggested to enhance permeability of epithelial tissues.^49^ The contributions of different types of Goblet cells to epithelial barrier functions and processes like antigen presentation are an important area of investigation.

TEER is dictated by the paracellular and transcellular routes of the epithelium, but for larger molecules the mucus layer is also thought to contribute to barrier properties of the intestinal mucosa. Due to our experimental setup, the ALI-VIP condition uniquely expresses a mucus layer. Our assays showed that permeability for smaller tracers was increased in LLI-VIP and ALI-NT, and highest in ALI-VIP conditions, while intermediate tracer permeability (40–70 kDa) was relatively comparable between conditions (Figure 5). Because of the complexity of the combined barrier function of the epithelium and mucus layer, it is challenging to determine the exact contribution of the mucus layer in the ALI-VIP condition. In addition, we acknowledge the possible downsides of the multilayered epithelium induced by ALI culturing. Hence, the choice of culture conditions with this system offers different options that can be tailored toward the specific research questions relating to mucus and barrier functions. For example, studies interrogating cancerous growth would benefit most from the ALI cultures, while research focused on intestinal epithelial barrier would benefit most from LLI conditions. Lastly, interrogations of mucus secretion, and bacterial-mucus interactions require a secreted mucus layer, which is unique to the ALI-VIP condition. In this latter category, we observed that commensal *L. plantarum* bacteria associated with the apical mucus layer at the Jacalin level, while pathogenic *Salmonella* bacteria invaded beyond the mucus layer (Figures 6, 7, 8). Therefore, it seems that the Caco-2 the ALI-VIP cultures provide a suitable platform with biomimetic features to investigate bacteria–mucus interactions. For example, it allows investigations into virulence factors that enable pathogens to pass the mucus layer and invade the intestinal epithelium.

While Caco-2 cells are easier and cheaper to culture than primary models, the downside of Caco-2 cells is their cancerous origin that may result in altered processes compared to primary non-transformed epithelium. ALI culturing of the Caco-2 cells induced growth of a multilayered epithelium, which perhaps is a feature linked to the cancerous origins of the cell line. In the intestine, the epithelium is single-layered, and perhaps the multilayered aspect of our ALI-VIP cultures affects the organization of cellular junctions and thereby epithelial permeability. In the LLI-VIP condition, the Caco-2 cultures remained single-layered, but under these conditions, no significant mucus secretion was achieved (Figure 1a, Figure S1b,c). We also observed differences in villin staining at the apical surface, perhaps due to experimental variation, leading to differences in differentiation state of the tissues (Fig. S1a and S1b,c). Hence, the choice of culture conditions with this system offers different options that can be tailored toward the specific research setting. For example, studies interrogating cancerous growth would benefit most from the ALI cultures, while research focused on intestinal epithelial barrier would benefit most from LLI conditions. Lastly, interrogations of mucus secretion and bacterial-mucus interactions require a secreted mucus layer, which is unique to the ALI-VIP condition. In recent years, *in vitro* primary intestinal models, including colonoids, enteroids, and gut-on-a-chip cultures, have emerged that produce MUC2 and have a functional mucus layer.^7,8^ These cultures are very promising but are costly, and different lines show variable proliferation, differentiation, and barrier formation. Another challenge is to recreate the anaerobic conditions of the intestinal tract to allow physiological studies with obligate anaerobic intestinal bacteria. Anaerobic conditions have been created by capping Transwells^11^ or in more complex systems such as the gut-microbiome (GuMI) physiome platform.^50^ The latter was developed to allow cocultures with oxygen-sensitive intestinal bacteria, and some MUC2-positive cells were visible in these cultures, but a secreted mucus layer was lacking. In addition to the Transwell culture systems and GuMI platform, *in vitro* multi-compartment simulators TIM-1, TIM-2, and SHIME offer the possibility to tailor conditions in different compartments, choose the microbial ecosystem, and mimic different intestinal environments, including pathogen infection.^51,52^ These simulators consist of multiple connected reactors and pumps to simulate the complexity of the human GI tract. SHIME has also been adjusted to include a mucus compartment (M-SHIME).^53^ In conclusion, several intestinal models are currently available that allow manipulation of different compartments or aspects of the bacteria–mucus–host interplay. An important challenge for the future remains the development of reproducible primary intestinal cultures with a functional mucus layer under physiological conditions. For applications that require high reproducibility and low costs, we are here presenting a novel Caco-2-based intestinal barrier model that can be used in Transwell and gut-on-a-chip applications. With its robust mucus layer, the model allows in-depth *in vitro* investigations into intestinal epithelial barrier properties and bacteria-mucosa interactions. We anticipate that our model will contribute to research into ulcerative colitis, Crohn’s disease, and leaky gut syndrome in which the interplay between microbiota, mucus, and inflammation plays a pivotal role in disease outcome.

## Materials and methods

### Cell culture

The parental human colorectal epithelial Caco-2 cell-line was purchased from ATCC. These Caco-2 cells were subcultured under low-glucose conditions for extended time leading to the Caco-2 cell-line used for all experiments in this study (Caco-2-MUC). Experiments have been performed in labs A (Figures 1a,b, 3 , 4 a,d, 5a,b, 5e,f, S1a, S2, S3), B (Figures 1c, 4 b,c, 5 c,d,g-h, 6, 7 , 8, S1b-d), and C (Figures 2, 5i,j), with similar protocols. The reproducibility of mucus production by the Caco-2 ALI-VIP cultures was high despite some differences in the methods between the labs. We feel that this shows that our method is robust, and therefore, we have made an effort to describe the methods used in the different labs. The Caco-2-MUC cell-line was routinely cultured in a 10 cm dish at 37°C in 6% CO_2_ (lab A) or T75 flasks at 37°C in 10% CO_2_ (lab B). Culture medium was composed of low glucose Dulbecco’s modified Eagle’s medium (DMEM) (Sigma) supplemented with 10% fetal bovine serum (FBS), 2 mm L-glutamine, 100 UI/ml penicillin, and 100 µg/ml streptomycin (lab A) or DMEM with pyruvate and glutamax (Thermo Fisher) with 10% FBS (lab B). Cells were seeded with 3.3E4 cells per 150 µl culture medium on rat-tail-collagen-I coated polyester Transwell inserts with 0.4 µm pores (Corning) (lab A) or polyethylene terephthalate (PET) tissue culture inserts with 0.4 µm pores from Sarstedt (lab B) and grown to confluency for 3 d followed by differentiation for 14 days (lab A) or 11 d with supplementation of 100 UI/ml penicillin and 100 ug/ml streptomycin (lab B) under four different culture conditions. In liquid–liquid interface non-treated conditions (LLI-NT), medium was refreshed in both apical and basolateral compartment without additional stimuli. In liquid–liquid interface VIP conditions (LLI-VIP), medium was refreshed in the apical compartment, and medium with 330 ng/ml vasoactive intestinal peptide (VIP) (#AS-22872; AnaSpec) was added to the basolateral compartment starting on day 3. In air–liquid interface non-treated conditions (ALI-NT), medium was removed from the apical compartment on day 3, and medium without any additional stimuli was added to the basolateral compartment. In air–liquid interface VIP conditions (ALI-VIP), medium was removed from the apical compartment on day 3, and medium containing 330 ng/ml VIP was added to the basolateral compartment. For all culture conditions, the compartments with medium with and without VIP were refreshed every 1–3 d, depending on experimental design. Medium refreshments were performed carefully with a pipette to avoid removal of potentially secreted mucus.

### RNA isolation and quantitative PCR (qPCR)

After 14 d of differentiation, total RNA was extracted from Caco-2 cultures using the RNAeasy isolation kit (Qiagen) or NucleoSpin RNA isolation kit (MACHEREY-NAGEL). RNA purity and concentration were measured with the NanoDrop 2000 spectrophotometer (Thermo Fisher Scientific). A 500 ng isolated RNA was reverse transcribed into cDNA using the iScriptTM cDNA Synthesis Kit (Bio‐Rad). mRNA expression was assessed by quantitative real-time PCR (qPCR) using SYBR® Select Master Mix (Thermo‐Scientific) in combination with the CFX384 Touch (Bio‐Rad). mRNA expression was normalized to the housekeeping gene ACTB. Primers used were MUC2_qFW1 (GCTGCTATGTCGAGGACACC), MUC2_qRV1 (GGGAGGAGTTGGTACACACG), ACTB_qFW1 (TTGTTACAGGAAGTCCCTTGCC) and ACTB_qRV1 (ATGCTATCACCTCCCCTGTGTG).

### RNA-sequencing and data analysis

Total RNA from treated and non-treated Caco-2 cells differentiated for 14 d was isolated using the QiaSymphony SP and the QIAsymphony RNA Kit (Qiagen). A 100 ng of total RNA was used for mRNA library preparation using the TruSeq Stranded mRNA kit (Illumina). Quality control was performed by the Useq sequencing facility, which resulted in the removal of some samples that were not of sufficient quality. Sequencing was performed in the Nextseq2000 platform (Illumina, Paired-end, 2 × 50bp reads). RNA sequencing data analysis was performed according to a standardized RNA sequencing analysis pipeline. In short, all samples were subjected to and passed quality control using FastQC (version 0.11.9). Subsequently, sequencing reads were mapped to the human genome (assembly GRCh38.109) using STAR RNA aligner (version 2.7.10b).^54^ In addition, raw and processed sequencing files were evaluated by MultiQC (version 1.13 (8dd46e3). FeatureCounts was used to count the mapped reads (version 2.0.3). For DEG analysis, the mapped reads were analyzed using R (RStudio 2023.03.1 + 446) package DEseq2 (version 1.34.0) for differential expression genes, and FDR-corrected values of *p* < 0.05 were considered statistically significant. PCA data was generated using DEseq2 after batch effect correlation using limma package (3.48.3) and plotted using ggplot2 (3.4.2). Gene set enrichment analysis was done using clusterProfiler (version 4.2.2) and gProfiler (version e109_eg56_p17_1d3191d) with multiple testing correction method using 0.05 as significance threshold. Heatmaps were plotted using ComplexHeatmaps (version 2.10.0). Raw and processed RNA-sequencing data from this study are available on Gene Expression Omnibus under accession number GSE283451 . RNAseq data from selected genes were analyzed with normalized counts, transformed when not normally distributed, and plotted using Prism GraphPad (v10.1.1). Applied statistical tests are stated in the legends of the figures.

### Immunoblotting

Caco-2 cells were differentiated in 24-well tissue culture inserts for 11 d. Cells were dissociated for 5 min with pre-warmed trypsin-EDTA (Gibco) and detached using a pipet tip. Cells were transferred to tubes and pelleted by centrifugation. The pellet was resuspended in 200–350 µl 50 mm Tris with 150 mm NaCl pH 7.4 (TBS) with 1% SDS, HALT protease inhibitor cocktail (#87786, Thermo Fisher), and 1 µl benzonase nuclease (Sigma) and incubated for 15 min at 37°C. Total protein concentration was determined by the BCA protein assay (Pierce) and normalized to a BSA standard curve (Pierce). 3× Laemmli sample buffer was added, and samples were heated to 95°C for 5 min before loading. To visualize MUC2, 10 μg total protein was loaded per lane on a 1% agarose gel containing 0.1% SDS, run in TAE buffer (40 mm Tris, 20 mm acetic acid, 1 mm EDTA) with 1% SDS for 2 h at 100 V. Proteins were transferred to a PVDF membrane (Immobilon 0.2 µm, Millipore) overnight in transfer buffer (600 mm NaCl, 60 mm sodium citrate) at room temperature. To visualize MUC13, 30 μg total protein was loaded per lane on a mucin SDS-PAGE (5% acryl/bisacrylamide gel without stacking gel), run in mucin running buffer (192 mm boric acid, 1 mm EDTA, 0.1% SDS, adjusted to pH 7.6 with Tris) for 2 h at 25 mA. Proteins were transferred to a PVDF membrane with 0.2 µm pores (1704156, Bio-Rad) with a semi-dry transfer (Trans-blot Turbo system, Bio-Rad) for 10 min at 1.3 A per gel. To visualize all other proteins, 10–20 µg protein was loaded on 12% SDS-PAGE with a 4% stacking gel, run in Tris-glycine SDS running buffer (Novex) at 100 V through the stacking gel and 200 V through the running gel. Proteins were transferred to Bio-Rad PVDF membranes for 7 min at 1.3 A per gel (Trans-blot Turbo system, Bio-Rad). All membranes were blocked with 4% BSA in TSMT (20 mm Tris, 150 mm NaCl, 1 mm CaCl_2_, 2 mm MgCl_2_ adjusted to pH 7 with HCl and 0.1% Tween 20) for 1 h at RT and incubated overnight at 4°C with primary antibody in 1% BSA in TSMT. Blots were washed for 3 × 10 min in TSMT followed by incubation with secondary antibody in 1% BSA in TSMT for 1 h at RT. Blots were washed 2 × 10 min in TSMT and 2 × 10 min in TSM and developed with Clarity Western ECL substrate (Bio-Rad) and imaged with the ChemiDoc MP imaging system (Bio-Rad) and quantified in ImageJ (1.53c).^55^ Primary antibodies used were α-MUC2 (1:1000, #662, in-house generated rabbit polyclonal), α-MUC13 (1:1000, #ab235450, Abcam), α-MUC17-C1 (1:1000, a kind gift of Thaher Pelaseyed, University of Gothenburg), α-claudin-1 (1:1000, #51-9000, Thermo Fisher), α-claudin-3 (1:1000, #34-1700, Thermo Fisher), α-claudin-4 (1:1000, #32-9400, Thermo Fisher), α-ZO-1 (1:1000, #ab216880, Abcam), α-GAPDH (1:1000, #G9545, Sigma), α-β-actin (1:1000, #bs-0061 R, Bioss), StcE-E447D-his probe (5 μg/ml, in-house recombinant production as described in 56). Secondary antibodies used were HRP-conjugated α-rabbit IgG antibody (1:5000, #A4914, Sigma), HRP-conjugated α-mouse IgG antibody (1:5000, #A230, Sigma), and HRP-conjugated His-probe (1:5000, #15165, Thermo Fisher).

### Immunofluorescence microscopy

In lab A (Figures 1, 4, 5), after differentiation, cells were fixed with methacarn (60% methanol, 30% chloroform, 10% glacial acetic acid) for 2 h at room temperature. Afterwards, cells were carefully washed with 100% methanol and rehydrated with decreasing concentrations of ethanol for 10 min each (96%, 90%, 80%, 70%, 60%, 40%, and 20%) in Hank’s balanced salt solution (HBSS). Cells were permeabilized, and nonspecific binding sites were blocked for 1 h with permeabilization/blocking (PB) buffer (2% BSA and 0.3% Triton X-100 in HBSS). Cells were incubated with primary antibodies diluted in PB buffer for 1 h at RT, washed 3× for 5 min with PB buffer, and incubated with secondary antibodies diluted in PB buffer for 1 h at RT. Then, the cells were washed 3× for 5 min with PB buffer and once with aquadest. Membranes were cut from the Transwell inserts and mounted on object glasses in between two drops of Prolong Gold antifade mountant (Thermo Fisher) and a coverslip. Finally, images were collected on a Leica SPE-II confocal microscope using a 40× or 63× objective (NA 1.3, HCX PLANAPO oil) controlled by Leica LAS AF software with default settings to detect DAPI, Alexa Fluor-488, Alexa Fluor-568, and Alexa Fluor-594. In lab B (Figures 6, 7, 8), after differentiation, cells were fixed with Carnoy’s fixative (60% ethanol, 30% chloroform, 10% glacial acetic acid) for 2 h at room temperature. Cells were washed with 100% ethanol and re-hydrated with 80% ethanol for 15 min. Antibody incubations and washes were described as above, but in blocking buffer (2% BSA in Dulbecco’s phosphate buffered saline; DPBS, Sigma, #D8537). Samples were mounted in Prolong Diamond antifade mountant (Thermo Fisher) as described above. Images were collected on a spinning disk Olympus SpinSR10 system equipped with a Yokogawa W1-SoRa spinning disk mounted on a I×83stand with an ORCA Flash 4.0 camera (Olympus, Leiderdorp, the Netherlands). The system was run in confocal mode using 40× oil objective (UPLXAPO, NA1.4) or 100× oil objective (UPLXAPO, NA1.45). Z slices imaged at 40× were 0.75 µm, and at 100× were 0.15 µm or 0.23 µm distance to visualize the entire cell layers, respectively. Fluorescence was recorded upon sequential excitation by the lasers (Coherent, OBIS) with appropriate emission filters and exposure time. The main dichroic mirror was a quadband (D405/488/561/640 nm). Orthogonal views were displayed in cellSens software (Olympus). Separation of the DAPI signal between bacteria and nuclei was optimized using constrained iterative deconvolution (20 iterations) on the DAPI image. Single-color images from MUC2, JAC, and deconvoluted DAPI were imported into ImageJ and displayed as composite images. Primary antibodies used are α-MUC2-AF647 or α-MUC2-AF488 (1:100, #sc -515,032, Santa Cruz), Jacalin-Fluorescein (1:100, #FL-1151-5, Vector lab) or Jacalin-biotin (1:250, #B-1155-5, Vector lab), α-ZO1 (1:100, #ab216880, Abcam), α-MUC13 (1:100, #ab235450, Abcam), α-Occludin (1:75, #sc -17,664, Santa Cruz), α-Villin (1:50, #610358, BD Biosciences), and DAPI (1:1000, #D9542, Sigma-Aldrich). Secondary antibodies used are goat α-rabbit IgG Alexa Fluor-488/568 (1:600, #A11034 and #A11031, Life Technologies), goat α-mouse IgG Alexa Fluor-488 (1:600, #A11029, Life Technologies), and streptavidin Alexa Fluor-568 (1:100, #S11226, Thermo Fisher).

### Gut-on-a-chip model and immunofluorescence microscopy

MidiKROS polyethersulfone (PES) hollow fiber membranes (HFMs, 0.2 μm pore size, 500 μm outer diameter, 300 μm inner diameter) were mounted in a 3D polylactide chamber, resulting in a fiber length of 2 cm inside the chamber. The bottom part of the chamber was sealed with a glass cover slip (Menzel-Gläser, Braunschweig, Germany) using Loctite EA *M*-31CL glue. Steel wire was inserted in the HFM to facilitate the insertion in the inner compartment of the chip. The communicating channels with the side reservoirs were sealed with GI-MASK Automix biocompatible glue (Coltene, Lezennes, France) to prevent leakage. Chambers were then sterilized overnight with UV light as well as with 70% (v/v) EtOH for 30 min. HFMs were subsequently coated with a biomimetic coating consisting of L-3,4-di-hydroxy-phenylalanine (L-Dopa, 2 mg/mL in 10 mm Tris buffer, pH 8.5) for 5 h at 37°C and 5% CO_2_ followed by coating with human collagen IV (25 µg/mL in PBS) for 2 h at 37°C and 5% CO_2_. Caco-2 cells were seeded inside the chambers at a density of 1 × 10^6^ cells per chamber and grown to confluency in LLI condition for 5–7 d, followed by differentiation in LLI-NT, LLI-VIP, ALI-NT, and ALI-VIP conditions for 14 d at 37°C with 5% CO_2_ on a 2-dimensional rocker at speed 10° per minute. Culture medium was refreshed every day. The apical compartment volume was 1 mL and the basal compartment volume 1,5 mL per side reservoir. After differentiation for 14 d, HFMs were detached from the chip system and fixed and rehydrated as described above. Cells on the HFMs were then permeabilized, and nonspecific binding sites were blocked for 1 h with PB buffer. Staining for immunofluorescence with α-MUC2-AF594 antibody (1:1000, #sc -515,032, Santa Cruz) and DAPI (1:1000, #D3571, Thermo Fisher) diluted in PB buffer was performed for 30 min at room temperature. HFMs were washed twice with PB buffer, followed by a washing step with MilliQ. The fibers were then dried and placed on a drop of Prolong gold solution (Thermo Fisher) on a WillCo-dish glass bottom dish, and another drop of mounting solution was placed on top of the HFM. Images were collected on a Leica DMi8 confocal microscope controlled by Leica LAS AF software. Images were processed using ImageJ software.

### Trans epithelial electrical resistance (TEER) measurements

Caco-2 cells were seeded on 24 well Transwell inserts and grown to confluency for 3 d. Trans epithelial electrical resistance (TEER) was measured on days 0, 3, 7, 10, and 14 after confluence with an EVOM2 epithelial Volt/Ohm meter (World Precision Instruments). Before measuring, fresh medium was added to both compartments, and the temperature was allowed to equilibrate. An empty Transwell was taken along as a blank, which was subtracted from all TEER values. After measuring, the medium was aspirated from the apical compartment in the ALI conditions, and VIP-conditioned medium was added to the basolateral compartment of the treatment conditions. The average of three distinct regions per well was calculated, and these values were then used to calculate the TEER in Ohm*cm^2^.

### Tracer assay

The basolateral medium of Caco-2 Transwell cultures was replaced for DMEM without phenol red (Thermo Fisher), with 10% FCS. On the apical side of all inserts, 150 µl filter-sterilized tracers dissolved in DMEM without phenol red, with 10% FCS, were added. Tracers were incubated for 2 h at 37°C, 10% CO_2_. Used tracers were Lucifer Yellow CH dipotassium salt (1 mg/ml, #L0144, Sigma), 4 kDa FITC-dextran (1 mg/ml, #46944, Sigma), 40 kDa FITC-dextran (1 mg/ml, #53379, Sigma), and 70 kDa FITC-dextran (1 mg/ml, #46945, Sigma). After incubation, 100 µl from the basolateral compartment was transferred to a 96-well plate, and fluorescence was measured using a FLUOstar Omega microplate reader (BMG Labtech). Lucifer yellow was measured at an excitation of 428 nm and an emission of 540 nm. FITC-dextran was measured with excitation at 492 nm and emission at 520 nm. An empty Transwell was taken along to calculate the percentage of paracellular permeability compared to complete permeability. For permeability assays with the gut-on-a-chip, a similar procedure was applied. The basolateral compartments of the gut-on-chip model were each loaded with 1,5 ml DMEM without phenol red (Thermo Fisher), with 10% FCS. On the apical side of the chip, 1 ml filter-sterilized tracers dissolved in DMEM without phenol red, with 10% FCS was added. Chips with tracers were incubated for 2 h at 37°C, 10% CO_2_, under rocking conditions as described above. Used tracers were 4 kDa FITC-dextran (1 mg/ml, #46944, Sigma), 40 kDa FITC-dextran (1 mg/ml, #53379, Sigma), and 70 kDa FITC-dextran (1 mg/ml, #46945, Sigma). After incubation, 100 µl from each basolateral compartment was transferred to a 96-well plate, and fluorescence was measured (excitation at 492 nm and emission at 520 nm) using a GloMax™ Discover Microplate Reader (Promega, Leiden, The Netherlands). Arbitrary fluorescence unit data were converted into percentage (%) and normalized to the positive control (chips without cells), which was assigned as 100% leakage.

### Bacteria and infection experiments

*Lactiplantibacillus plantarum* (strain ATCC-14917) was cultured overnight on MRS agar plates at 37°C. A single colony was picked and grown o/n in MRS broth, shaking at 37°C. OD_600_ of the overnight culture was measured. An OD_600_ of 1.0 contained about 9.7 × 10^7^ CFU/ml. We aimed to infect each well with 30 µl DMEM without FCS containing 1.2 × 10^7^ CFU. Before infection, bacteria were first stained with live CellTrace dye, CFSE (Invitrogen, C1157). To achieve staining, the required number of CFU was spun down, and the pellet was resuspended in DMEM without FCS containing 60 μM CFSE. The bacteria were incubated for 45 min at 37°C, 10% CO_2,_ followed by 3 washes with DMEM with FCS. At the last wash, the bacterial pellet was resuspended into DMEM without FCS. Before infection, the medium from the apical compartment of LLI cultures was removed. Bacteria were incubated with the Caco-2 cultures for 1 or 3 h at 37°C, 10% CO_2_. Then, cell layers were washed twice with DMEM without FCS and further processed for immunofluorescence microscopy. *Salmonella enterica* serovar Enteritidis (strain 90-13-706, CVI, Lelystad) was previously used for infection studied in our group^39^ and cultured overnight on LB agar plates at 37°C. A single colony was picked and grown overnight in LB broth, shaking at 37°C. Bacteria were subcultured by 1:1000 dilution in 25 ml fresh LB in a 250 ml Erlenmeyer, shaking at 37°C until OD_600_ of 1.0 was reached. An OD_600_ of 0.24 contained around 2.4 × 10^8^ CFU/ml. We aimed to infect each well with 30 µl DMEM with FCS containing 1.3 × 10^6^ CFU. Therefore, the required number of CFU was spun down, and the pellet was resuspended in DMEM with FCS. Before infection, the medium from the apical compartment of LLI cultures was removed. Bacteria were incubated with Caco-2 cells for 1 h at 37°C, 10% CO_2_. Then, cell layers were washed twice with DMEM with FCS and further processed for immunofluorescence microscopy. To generate a fluorescent Salmonella strain, *Salmonella enterica* serovar Enteritidis was transformed with plasmid pMW85 expressing GFP from a PpagC promoter (a kind gift from Dr Dirk Bumann.^57^ *Salmonella*-GFP bacteria were cultured on an LB agar plate or liquid medium with 100 μg/ml ampicillin. Infection experiments with the *Salmonella*-GFP strain were performed as described above. *Escherichia coli* (ETEC DSMZ-27503) infection was essentially performed as the *Salmonella* infection, with minor adjustments. Bacteria were subcultured 1:100 in 5 ml fresh LB in a 50 ml tube. Bacteria were grown to OD_600_ of 0.8. Infection was performed in DMEM without FCS. An OD_600_ of 1.0 contained around 1.0 × 10^9^ CFU/ml. We aimed to infect each well with 30 µl DMEM without FCS containing 1.2 × 10^7^ CFU.

### Image analysis

For quantification of *L. plantarum* adherence to the Caco-2 cultures, images were recorded at 40× (UPLXAPO oil NA1.4)) on the spinning disk and analyzed using Imaris software (v10.2). A script was developed and used for batch analysis. In brief, each image field was axially compressed to 0.32 µm before preprocessing. In image preprocessing, first, each field was background subtracted with a radius of 150 pixels, then deconvoluted settings: sharpening 4, 4 iterations, and 0.2 noise reduction. After deconvolution, baseline subtraction of intensity 100 was performed. Hereafter, each image was analyzed using the spot detection wizard, and distance to the nearest neighbor was measured with the following settings: Estimated XY Diameter = 0.750 µm, Estimated Z Diameter = 2.00 µm, Background Subtraction = true, [Filter Spots] of “Quality” above 35.0 and “Intensity Mean” above 1250. A detailed script can be made available upon request. The algorithm analyzed the number of bacteria and distance to the nearest bacteria per bacterium per image taken.

## Supplementary Material

Supplemental Material

## Data Availability

The authors confirm that the data supporting the findings of this study are available within the article and its supplementary materials. RNAseq data were generated at USeq, Center for Molecular Medicine, UMC Utrecht. Raw and processed RNA-sequencing data from this study are available on Gene Expression Omnibus under accession number PRJNA1076117. RNAseq-derived data are available within the article and its supplementary materials.
