# Supplementary material for: Development of a Caco-2-based intestinal mucosal model to study intestinal barrier properties and bacteria–mucus interactions
Source: Gut Microbes. 2024 Dec 23;17(1):2434685. doi: 10.1080/19490976.2024.2434685 (PMC11702969; doi:10.1080/19490976.2024.2434685)
Supplement: Supplemental Material [file KGMI_A_2434685_SM3704.zip › figures 7-S3.docx]

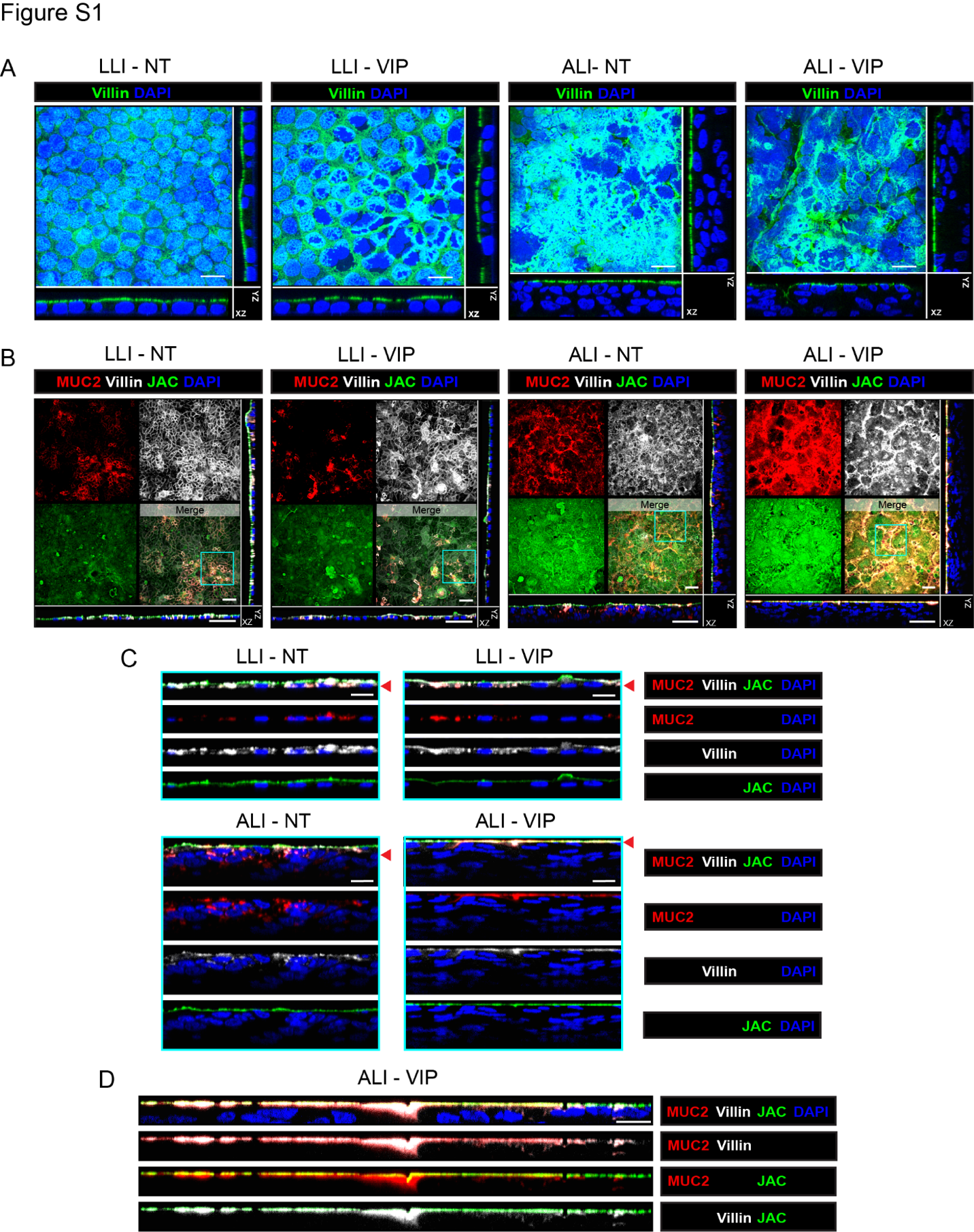


**Figure S1. Differentiation of Caco-2 cell under different culture conditions.**

(A) Confocal microscopy of confluent Caco-2 cultures grown on Transwell membranes in LLI and ALI conditions, not treated (NT) or treated with VIP. Cultures are stained for Villin (green) and nuclei (DAPI, blue). Maximum projections and orthogonal views are shown. White scale bars represent 5 μm. (B) Confocal microscopy of confluent Caco-2 cultures stained for MUC2 (red), Villin (white), Jacalin (green), and nuclei (blue) with enlarged orthogonal views to display the apical surfaces of the different cultures. White scale bars represent 50 μm. (C) Enlarged section of the XZ orthogonal views (cyan squares depicted in B) showing the localization of MUC2 (red) relative to the apical brush border stained by villin (white) in the four different culture conditions, white scale bars represent 10 μm. (D) Orthogonal view of ALI-VIP Caco-2 cultures visualizing (co)localization of MUC2, villin, and Jacalin, white scale bar represent 10 μm.


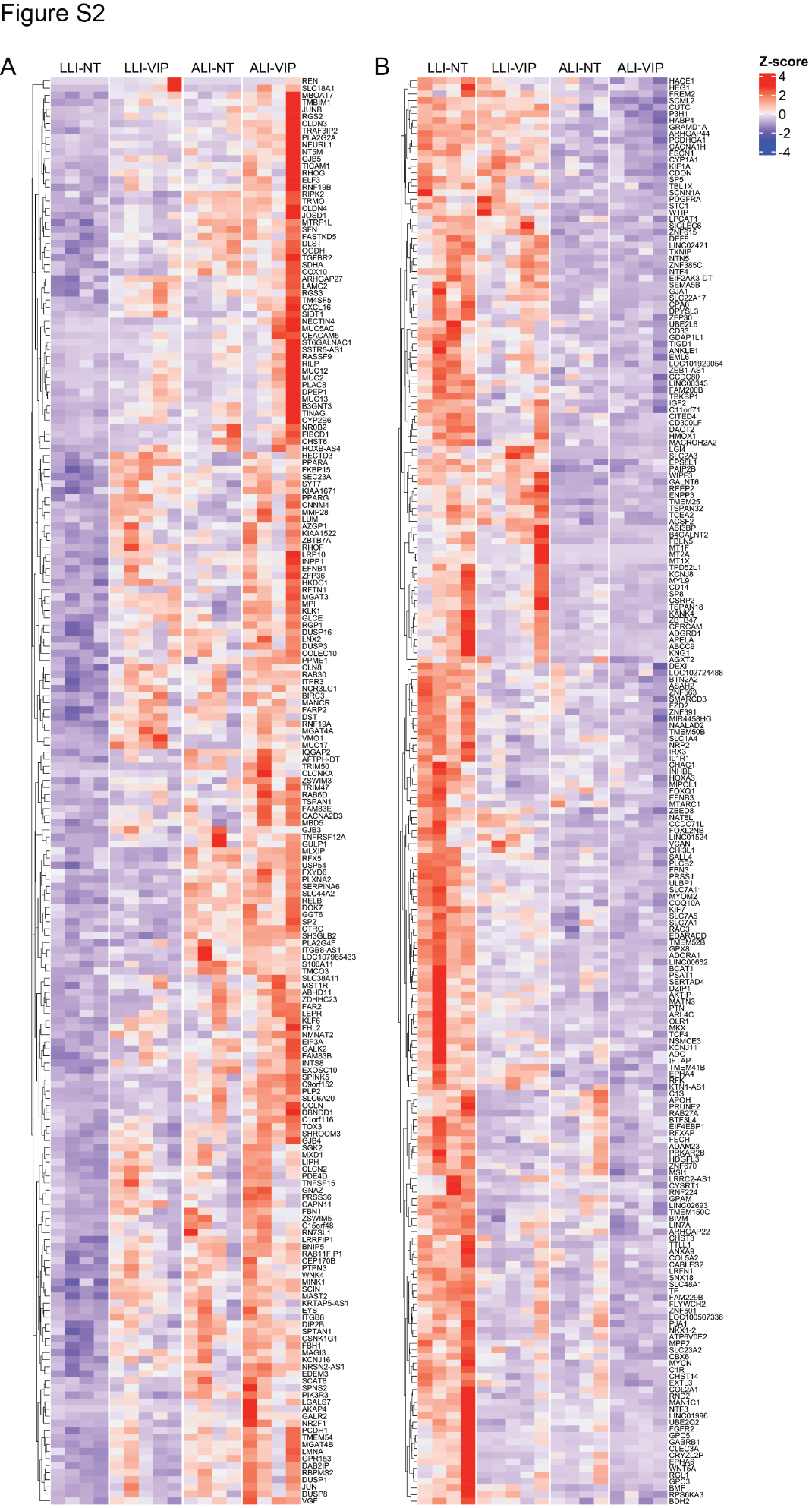


**Figure S2. Differentially expressed genes between LLI-NT and ALI-VIP conditions.**

(A) Heatmap of the 209 upregulated genes in the ALI-VIP condition compared to the LLI-NT condition with gene labels on the right. (B) Heatmap of the 243 downregulated genes in the ALI-VIP condition compared to the LLI-NT condition with gene labels on the right. See also supplementary table 1-5 for a full list of all DEGs between the different culture conditions. See also Figure 3B for a compressed heatmap of the same 452 DEGs between ALI-VIP and LLI-NT.


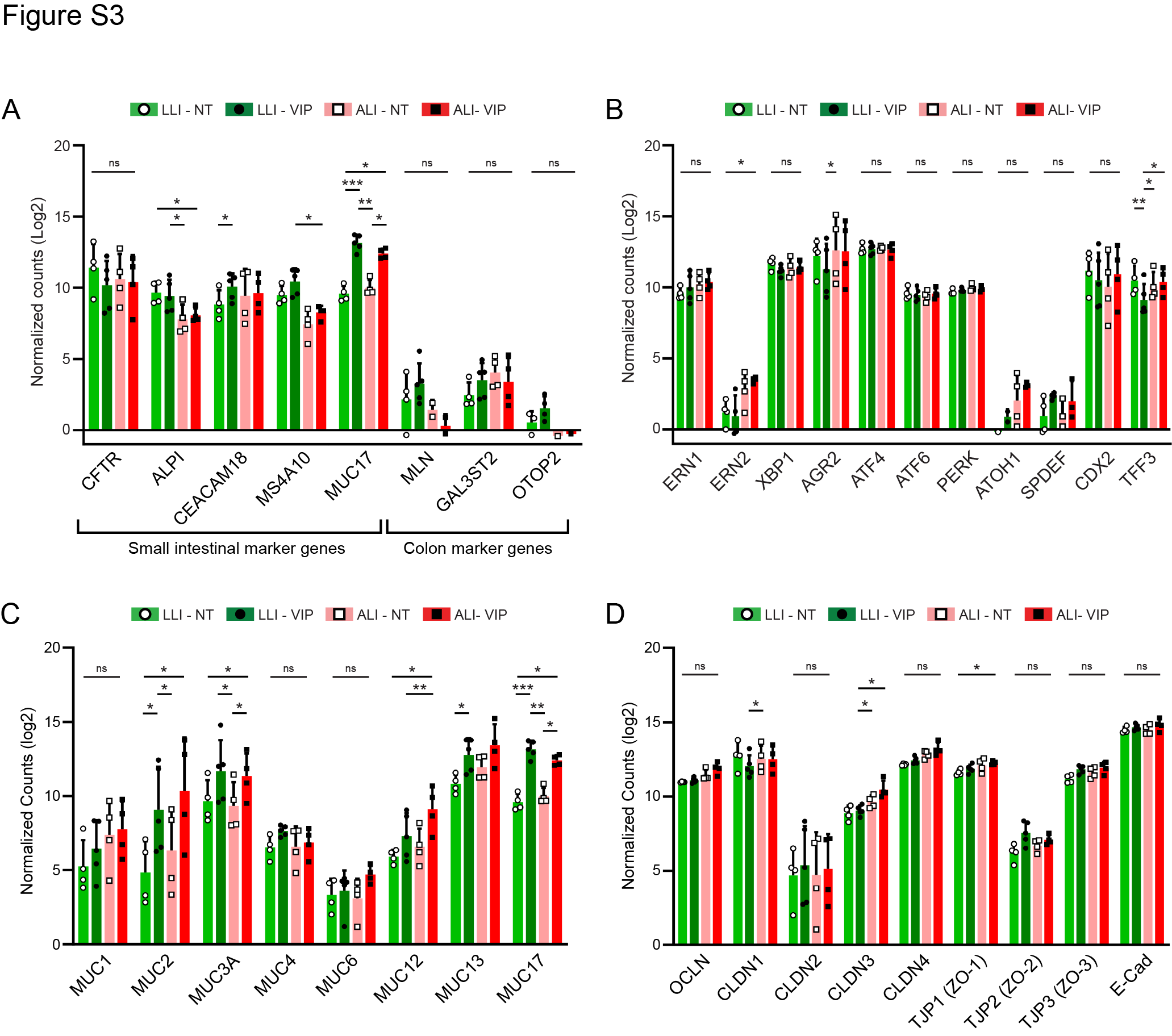


**Figure S3. Gene expression analysis of marker genes, transcription factors, mucin genes, and junction genes from the RNAseq dataset**

(A) Expression of small intestinal (CFTR, ALPI, CAECAM18, MS4A10, and MUC17) and colon marker genes (MLN, GAL3ST2, and OTOP2) in Caco-2 cells grown under LLI-NT, LLI-VIP, ALI-NT, and ALI-VIP conditions. (B) Expression of mucus-related transcription factors ERN1/IRE1α ERN2/IRE1β, XBP1, AGR2, ATF4, ATF6, PERK, ATOH1, SPDEF, CDX2, and TFF3 in Caco-2 cells cultured under LLI-NT, LLI-VIP, ALI-NT and ALI-VIP conditions. (C) Expression of mucin genes MUC1, MUC2, MUC3A, MUC4, MUC6, MUC12, MUC13 and MUC17 in Caco-2 cells grown under LLI-NT, LLI-VIP, ALI-NT and ALI-VIP conditions. (D) Expression of cellular junction genes OCLN, CLDN1, CLDN2, CLDN3, CLDN4, TJP1, TJP2, TJP3, and E-cadherin in Caco-2 cells grown under LLI-NT, LLI-VIP, ALI-NT and ALI-VIP conditions. For all graphs, normalized counts from the RNAseq data transformed for individual data points of the biological replicates were plotted. Statistical analysis for all graphs was performed by ordinary one-way ANOVA under mixed-effect analysis using GraphPad Prism software (v10.1.1). * p<0.05; ** p<0.01
